# A Bibliometric Analysis Review of the Top 50 Most Cited Articles on Incretin-Based Therapy for Weight Reduction and Diabetes Management

**DOI:** 10.7759/cureus.102500

**Published:** 2026-01-28

**Authors:** Ehab F Alsaygh, Abdullah M Aljarboa, Adel A Alhazmi, Abdullah S Alhazmi, Osamah A Alraddadi, Aseel A Althagafi, Mohmmed F Alhojele

**Affiliations:** 1 Family Medicine, Family Medicine Academy, Ministry of Health, Medina, SAU; 2 Family Medicine, Ministry of National Guard Health Affairs, Jeddah, SAU; 3 Obesity and Family Medicine, King Salman Bin Abdulaziz Medical City, Medina, SAU

**Keywords:** bibliometric, diabetes, dpp-4 inhibitors, glp-1 receptor agonists, incretin, liraglutide, obesity, semaglutide, weight loss

## Abstract

The introduction of incretin-based therapy has brought a significant transformation in the treatment of type 2 diabetes and obesity. It works by augmenting natural incretin hormones to enhance insulin secretion and suppress glucagon release in a glucose-dependent manner. The primary goal of this research was to identify the top 50 most cited articles in this field, explore the trends, recent developments and the future direction of the research in this field.

This is a bibliometric analysis. The subjects consisted of the 50 most cited scientific publications that were extracted from the Web of Science Core Collection on May 11, 2025. The research strategy used keywords related to incretin therapy, weight loss and type-2 diabetes.

The research approach consisted of a database search combined with a quantitative assessment of publication metrics. The articles we identified were published between 1999 and 2023. Statistical analysis used IBM SPSS Statistics for Windows, Version 31 (Released 2025; IBM Corp., Armonk, New York, United States) to calculate descriptive statistics including mean, standard deviation, median and frequencies for data summary.

The 50 articles received an average of 473.44 citations with a citation range between 188 and 3,045. The majority of publications appeared in Q1 journals with impact factors ranging from 2.2 to 98.4 and Diabetes Care (28%) and The Lancet (14%) being the most prominent journals. Thirteen countries contributed, predominantly the United States (56%), followed by Denmark (10%).

The analysis successfully identified the most influential literature, confirming the significant and growing impact of incretin-based therapy research. The field demonstrates a clear trend from GLP-1 agonists towards dual and triple receptor agonists, with research heavily published in high-impact journals and led by US-based, multi-author teams.

## Introduction and background

Incretin-based pharmacotherapy, spearheaded by glucagon-like peptide-1 receptor agonists (GLP-1 RAs), has transformed modern management of type 2 diabetes mellitus by lowering plasma glucose and producing clinically meaningful weight loss [1]. Mechanistically, GLP-1 receptor activation slows gastric emptying and suppresses appetite, resulting in consistent body-weight reduction [1]. Incretins are peptide hormones produced in the gut and released when nutrients are consumed, mainly glucagon-like peptide-1 (GLP-1) and glucose-dependent insulinotropic polypeptide (GIP), which promote glucose-dependent insulin secretion and contribute to postprandial insulin [1]. In addition to their effects on insulin, GLP-1 inhibits glucagon secretion, slows down gastric emptying, and enhances feelings of fullness, thus aiding in glycemic control and facilitating weight loss, while GIP provides additional insulin-promoting actions [1,2]. These biological mechanisms serve as the scientific basis for therapies involving incretins in the management of diabetes and obesity.

Evidence from clinical trials, as summarized in contemporary reviews, indicates that GLP-1 RAs are among the most effective available pharmacologic options for obesity management [3]. This is illustrated by the STEP program, where weekly subcutaneous semaglutide 2.4 mg achieved mean weight losses of 14.9-17.4% over 68 weeks in non-diabetic adults, as summarized in recent reviews [2].

Building on this success, dual agonists targeting GIP and GLP-1 receptors emerged. Early trials of LY3298176, tirzepatide’s precursor, showed dose-dependent glucose lowering and significant weight loss, supporting the potential of dual incretin "twincretin" therapy [4]. Tirzepatide, a GIP-biased agonist, further enhanced insulin secretion and broad metabolic control [5]. A 12-trial meta-analysis of 11,758 participants confirmed tirzepatide’s superior efficacy compared to placebo, insulin, and GLP-1 RAs [6].

Triple receptor agonism represents the next step. Retatrutide, a single-molecule GLP-1/GIP/glucagon agonist, demonstrated significant weight reduction and improved metabolic parameters without excess adverse events [7]. Survodutide, a dual glucagon/GLP-1 RA, reduced HbA1c and produced dose-dependent weight loss up to 8.7% at 16 weeks, with doses ≥1.8 mg once weekly achieving greater weight reduction than open-label semaglutide (up to 1.0 mg once weekly) over the same period [8].

These developments underscore a rapid, citation-rich expansion of incretin literature. Bibliometric studies have been used to analyze research trends in diabetes, obesity, and GLP-1 therapies [9,10]. However, a focused bibliometric evaluation of the most influential articles on incretin-based therapy for both weight reduction and diabetes management is lacking.

Mapping this growth is vital for clinicians, researchers, and policymakers to identify influential studies, investigators, journals, and shifting research priorities from single GLP-1 RAs to dual and triple agonist strategies.

This study will systematically identify and characterize the 50 most cited articles on incretin-based therapy for weight management and diabetes control. Nevertheless, there remains a notable gap in assessing the most cited incretin-based therapy articles, considered highly influential in diabetes and obesity medicine. Citation scoring also recognizes authors, countries of origin, journals, and journal impact factors (IFs) [11-13].

## Review

Methods & materials

Search Strategy

On May 11, 2025, we searched the Web of Science Core Collection to identify the most cited articles on incretin-based therapy for weight reduction and diabetes management. Retrieved records were exported on the same date for screening, ranking, and data extraction to ensure reproducibility of citation counts at the time of retrieval. The search strategy incorporated combinations of the following terms ("incretin-based therapy" OR "GLP-1 receptor agonist*" OR "GLP-1 agonist*" OR "glucagon-like peptide-1" OR "dual agonist*" OR "triple agonist*" OR "GIP/GLP-1" OR "GLP-1/GIP/glucagon")AND ("weight reduction" OR "weight loss" OR "obesity" OR "BMI" OR "body weight" OR "anti-obesity" OR "appetite suppression")AND ("type 2 diabetes" OR "diabetes management" OR "diabetes" OR "glycemic control" OR "HbA1c" OR "glucose control" OR "insulin resistance"). Only original research articles and reviews were included in the search. Bibliometric methods are widely accepted approaches for evaluating research impact, productivity, and knowledge structure within scientific fields and have been applied extensively in biomedical research [14,15]. Retrieved records were ranked according to citation frequency, and the top 50 most cited publications were selected. Through this procedure, the top 50 articles cited for incretin-based therapy for weight reduction and diabetes management were found for bibliometric analysis.

Study Selection

Titles, abstracts, and full texts were screened by two authors. Disagreements were resolved by arbitration from a third reviewer.

All records were ranked in descending order based on the total number of citations (Times cited) in Web of Science (total citations accumulated up to May 11, 2025). No ties were encountered at the inclusion threshold (i.e., between the 50th and 51st ranked records); therefore, no tie-breaking procedure was required. The 50 highest-cited articles meeting the eligibility criteria were included.

Inclusion Criteria

Articles published in English, peer-reviewed journal publications, those with a primary focus on incretin-based therapy (e.g., GLP-1 RAs and/or related incretin therapies) in relation to weight reduction and/or diabetes management, and studies involving human participants or human patient populations were included.

Exclusion Criteria

**Figure 1:**
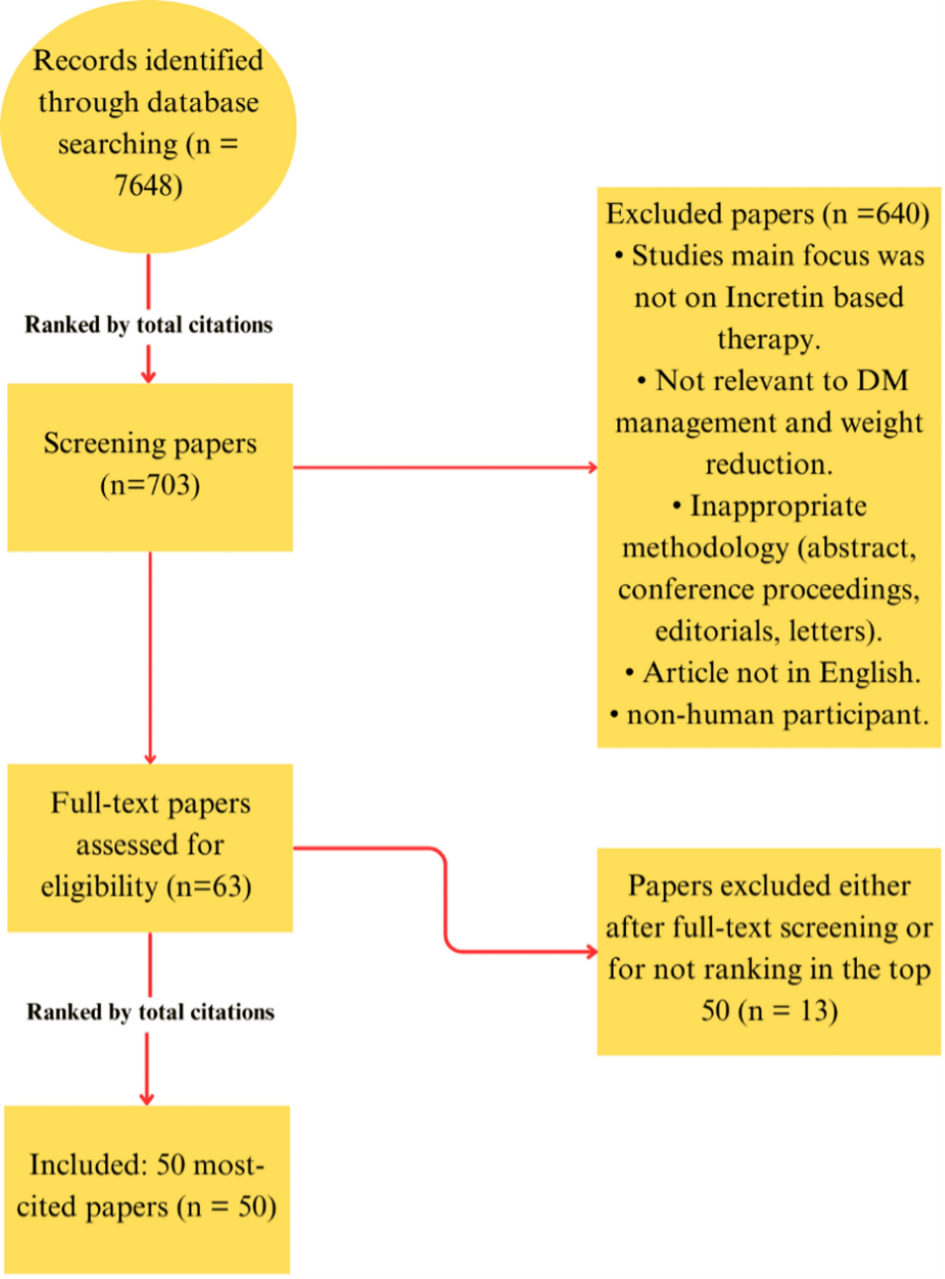
Flowchart of the methodology. DM: Diabetes mellitus

Non-peer-reviewed materials (conference proceedings, abstracts, posters, and similar formats), non-English publications, animal or in vitro studies, and publications not primarily addressing incretin-based therapy in the context of weight reduction and diabetes management were excluded (Figure 1).

Data Extraction and Management

Data were extracted from the included studies using a standardized data extraction sheet in Microsoft Excel (version 16.62). The following points were recorded from the included papers: Article title, authors, publication year, country of publication, journal, total number of citations, average number of citations annually, IF of the journal, and funding status.

Treatment of Missing Data

If any bibliometric field was missing from the exported record, we verified it through the journal website, Journal Citation Reports, or the database record; if still unavailable, it was reported as missing without imputation.

Statistical Analysis

Statistical analyses were descriptive. Continuous variables were summarized as mean ± standard deviation (SD) or median (interquartile range, IQR), and categorical variables as frequencies and percentages. No inferential statistical testing, meta-analysis, meta-regression, confidence intervals, or P-values were performed. Analyses were performed using IBM SPSS Statistics for Windows, Version 31 (Released 2025; IBM Corp., Armonk, New York, United States) for descriptive computations only.

Risk of Bias

A risk of bias assessment was not performed because this study is a bibliometric analysis focused on citation performance and publication characteristics.

Results

The bibliometric analysis explores the top 50 most cited articles on incretin-based therapy on weight reduction and diabetes management over the period 1999-2023.

Citation Metrics

The number of citations per article ranged from 188 to 3,045, with an average of 473.44. The most cited article garnered 3,045 citations, with an average annual citation rate of 43.94 (SD ±39.09). Journal IFs ranged from 2.2 to 98.4, with a mean of 36.45 (SD ±36.26).

Journal Distribution

Analysis of 50 studies shows diverse representation across 17 high-impact journals, indicating a strong scientific foundation. The most frequently cited journals included Diabetes Care (28%), The Lancet (14%), Diabetes Obesity & Metabolism (10%), Lancet Diabetes & Endocrinology (10%), and JAMA (6%), with others contributing 2-4% (Table 1). 

**Table 1:** Publication count, impact factor, and Q ranking of the journals.

Rank	Journal	No. Publications, n (%)	IF	Q Ranking
1	Diabetes Care	14 (28%)	14.8	Q1
2	Lancet	7 (14%)	98.4	Q1
3	Diabetes Obesity & Metabolism	5 (10%)	5.4	Q1
4	Lancet Diabetes & Endocrinology	5 (10%)	44.0	Q1
5	JAMA-Journal of the American Medical Association	3 (6%)	63.5	Q1
6	BMJ-British Medical Journal	2 (4%)	93.7	Q1
7	Journal of Clinical Endocrinology & Metabolism	2 (4%)	5.0	Q1
8	Molecular Metabolism	2 (4%)	7.0	Q1
9	New England Journal of Medicine	2 (4%)	96.2	Q1
10	American Journal of Physiology-Regulatory Integrative and Comparative Physiology	1 (2%)	2.2	Q2
11	BMJ Open	1 (2%)	2.4	Q1
12	Cell Metabolism	1 (2%)	27.7	Q1
13	Clinical Therapeutics	1 (2%)	3.2	Q1
14	Diabetes	1 (2%)	6.2	Q1
15	Diabetologia	1 (2%)	8.4	Q1
16	Frontiers in Endocrinology	1 (2%)	3.9	Q1
17	Nature Reviews Endocrinology	1 (2%)	31.0	Q1

Publication Year Distribution

**Figure 2:**
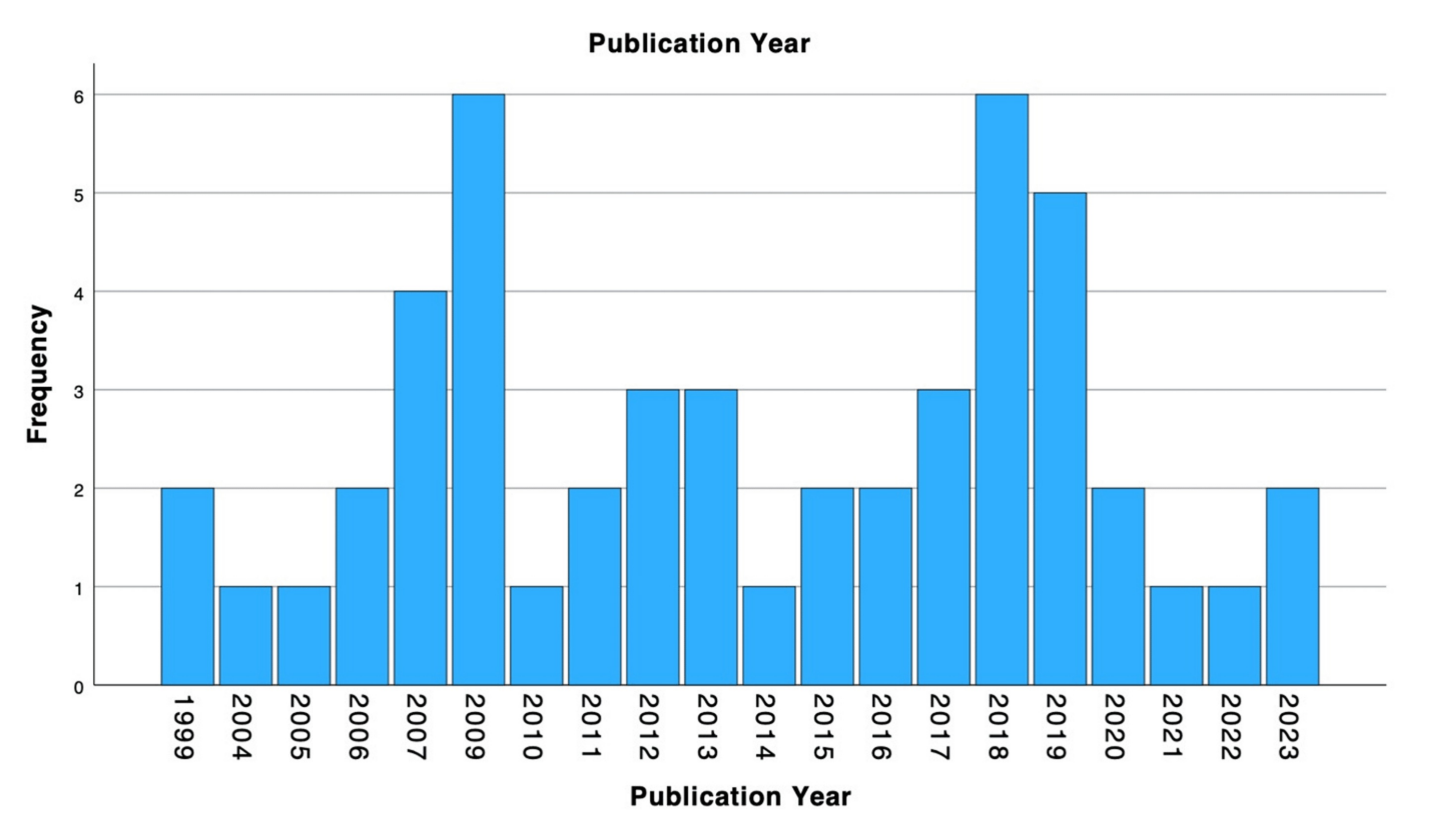
Publication years of the studies. The figure illustrates publication trends from 1999 to 2023, highlighting periods of increased research output in this field.

Distribution of the top 50 most cited articles on incretin-based therapy for weight reduction and diabetes management according to year of publication is shown in Figure 2.

Geographic Contribution

**Figure 3:**
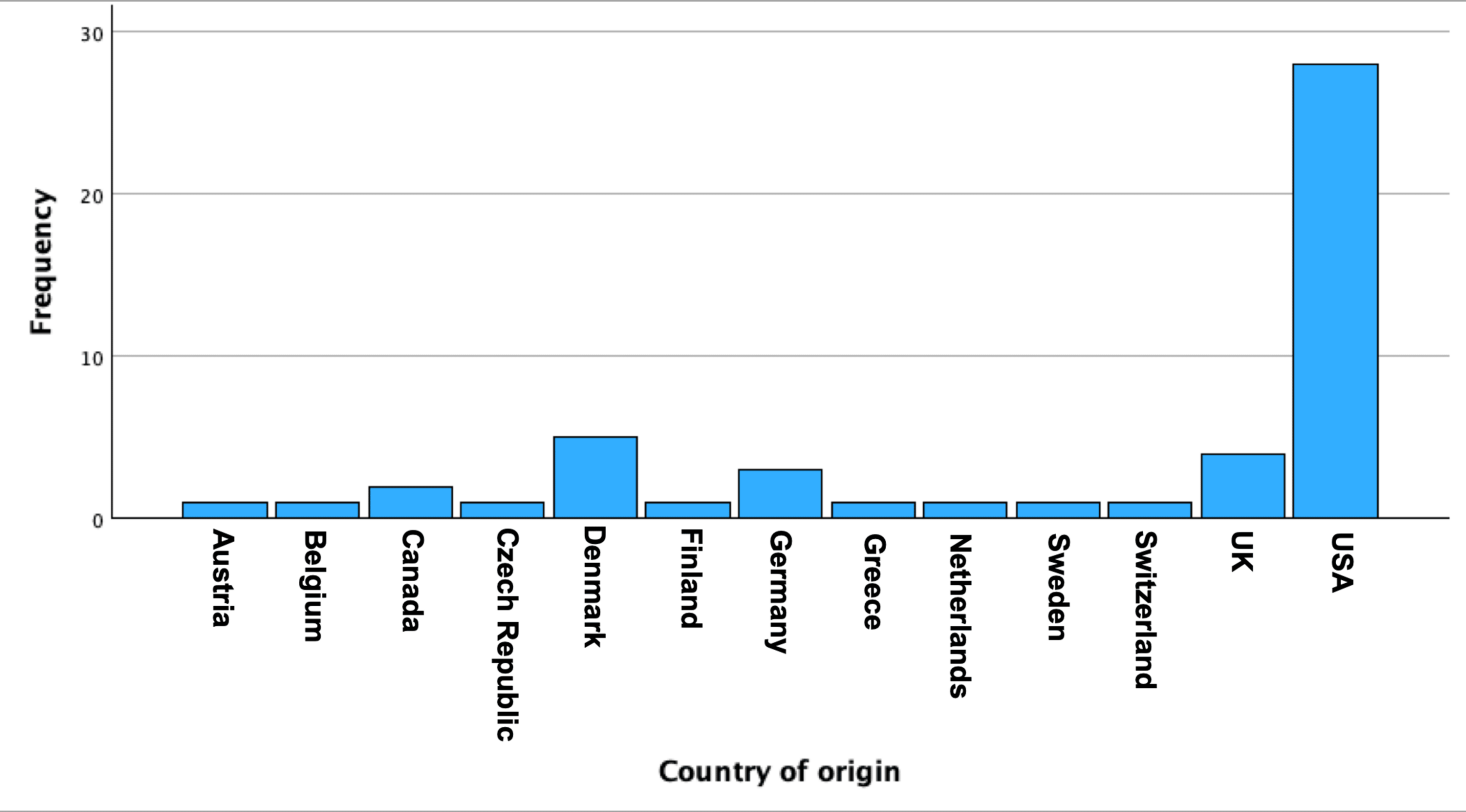
Geographic distribution of the top 50 most cited articles on incretin-based therapy for weight reduction and diabetes management. The figure shows the country of origin of the publications, with the United States contributing the largest proportion, followed by Denmark, the United Kingdom, and Germany.

Thirteen countries contributed to the publications, predominantly the United States (56%), followed by Denmark (10%), the United Kingdom (8%), and Germany (6%). Other European and North American countries contributed smaller proportions (2-4% each). (Figure 3).

Authorship Characteristics

The number of authors per study, shown in Table 2, ranged from 1 to 16, with a median of eight authors. The most common author count was 7 (18%), followed by 9 (14%) and 10 (12%). Studies with 5 to 10 authors accounted for 66% of the total (n = 50). 

**Table 2:** Author count across the included studies.

No. of authors	Frequency, N (%)
1	1 (2 %)
2	4 (8 %)
3	2 (4 %)
5	5 (10 %)
6	2 (4 %)
7	9 (18 %)
8	5 (10 %)
9	7 (14 %)
10	6 (12 %)
11	4 (8 %)
12	3 (6 %)
13	1 (2 %)
16	1 (2 %)
Total	50 (100 %)

Funding Reporting

Out of the analyzed articles, 16 (32%) articles did not report any funding sources.

Discussion

Our bibliometric analysis highlights the top cited articles on incretin-based therapy for weight loss and DM management over the period 1999-2023; most of these papers were published in high-impact journals, reflecting the significance strength and clinical relation of this field.

Geographically, many of these articles originated in the United States, accounting for over half of the top-cited articles. This dominance may be due to the country's strong research funding and the early adoption of incretin-based therapies in clinical practice. Other contributing countries included Denmark and the United Kingdom.

When compared with previous bibliometric studies in diabetes, our findings demonstrate a similar concentration of publications in Western countries and high-impact journals. However, what we're seeing here is a clear shift. Earlier research often centered on insulin or glucose monitoring. This study reveals a shift toward more recent pharmacologic innovations with double benefits in glycemic control and weight reduction. The findings reinforce the important role of incretin-based therapies in reshaping modern diabetes care and highlight areas for future research.

From our results in regard to the journals of published studies, it was noted that the journals dominating the field were mainly Diabetes Care, The Lancet, Diabetes Obesity & Metabolism, and Lancet Diabetes & Endocrinology.

The mean count of citations was 473, which is high, and on average, the annual citation count was 44. Citation counts are frequently used as measures of scholarly impact and influence within the scientific community, despite the fact that they do not directly reflect scientific quality [16].

Most of the journals were Q1-ranking, with IFs ranging from 2.2 to 98.4 (mean 36.45). This corroborates broader bibliometric findings that highly impactful studies typically appear in highly impactful journals [17].

It can also be noted that studies that gained the most attention were published in related fields such as Diabetes Care or in broad field journals such as The Lancet.

Authorship analysis demonstrated a collaborative research pattern, with a median of eight authors per study and two-thirds of articles involving five to 10 authors [17,18]. This reflects the complex study designs that are typical in incretin clinical trials.

Nearly one-third of the studies lacked funding disclosures, which suggests either independent efforts or insufficient reporting, as sponsorship can affect the study design or interpretation. Notably, among those disclosing funding, Novo Nordisk, with 17 mentions, Eli Lilly, with 11 mentions, and Merck, with five mentions, were the most prominent sponsors, highlighting their substantial involvement in incretin-based therapy research.

The considerable contribution of company sponsors reflects their role in advancing incretin-based therapy research. However, it also highlights the importance of incorporating independent, real-world data into these findings. Real-world evidence can provide valuable insights into the effectiveness and safety of these therapies in everyday clinical practice, ensuring a more balanced and comprehensive understanding beyond controlled trial settings [19,20]. Future bibliometric evaluations should examine the sources of funding to better understand bias potential [21].

Recommendation and Limitations

This bibliometric analysis faced limitations mainly in the focus on English-language articles, which potentially overlooked studies published in other languages.

Future bibliometric studies should address these limitations by incorporating non-English literature to ensure a more comprehensive and inclusive representation of the research area.

The IF should not be relied on as the sole measure of a journal’s quality or reputation; quartile rankings should also be considered to provide a more balanced and accurate evaluation [22].

Citation counts are inherently time-sensitive, as recently published articles have had less opportunity to accrue citations [22]. Moreover, citation metrics may be influenced by several external factors, including journal visibility, open-access status, self-citation practices, and potential journal bias [23]. Importantly, a high citation count may reflect widespread discussion or controversy rather than scholarly endorsement and therefore does not necessarily indicate the quality or novelty of the research findings.

## Conclusions

This bibliometric analysis highlights the most important papers on incretin-based weight loss and diabetes treatment, highlighting the field's explosive growth over the previous 20 years. The prevalence of U.S.-based research and publications in prestigious journals like The Lancet and Diabetes Care illustrates how important incretin-related developments are on a global scale.

The dual burden of obesity and hyperglycemia is being addressed by emerging trends that clearly demonstrate a shift from GLP-1 monotherapy to more intricate dual and triple agonist approaches. Future studies should concentrate on underexplored areas such as long-term safety, comparative effectiveness between newer agents, and the role of incretin therapies in non-diabetic populations.
